# Revolutionizing Eco-Friendly Leather Production: A Freeze-Thaw and Liquid Fermentation Approach with Fungal Mycelium

**DOI:** 10.3390/jof11040326

**Published:** 2025-04-19

**Authors:** Linxin Song, Yuxin Liu, Shijun Xiao, Xiaohui Yuan, Xuerong Han

**Affiliations:** International Cooperation Research Center of China for New Germplasm Breeding of Edible Mushrooms, Jilin Agricultural University, Changchun 130118, China; slinxin@yeah.net (L.S.); 3042134612@gg.com (Y.L.); xiaoshijun@jlau.edu.cn (S.X.); yuanxh@gooalgene.com (X.Y.)

**Keywords:** freeze-thaw dehydration, ice crystals, liquid fermentation, fungal mycelium, mycelium leather

## Abstract

The environmental impact and resource demands of traditional leather manufacturing have driven the search for sustainable alternatives. Fungal mycelium leather, recognised for its eco-friendly and renewable characteristics, has emerged as a promising option. This study established a cyclic freeze-thaw dehydration protocol for preparing mycelial leather using *Ganoderma* mycelium produced through liquid fermentation. By precisely controlling the fermentation parameters (pH 5.5, 150 rpm agitation, 28 °C), the liquid fermentation process ensures uniform mycelial growth, which is critical for subsequent structural enhancement during freeze-thaw cycles. After three freeze-thaw cycles were performed at −15 °C, uniformly distributed ice crystals facilitated effective water removal, achieving a minimum moisture content of 47.6%. The optimized freeze-thaw process produced membranes with a tensile strength of 6.22 MPa and elongation at break of 18.92%, demonstrating high mechanical performance. The freeze-thaw process was demonstrated to enhance structural integrity and mechanical properties while offering reduced energy consumption compared to conventional dehydration methods. This research provides a theoretical foundation and technical guidance for optimising fungal mycelium leather production and contributes to the development of sustainable bio-based materials for industrial applications.

## 1. Introduction

Traditional leather, which is widely used in the fashion, furniture, and automotive industries, has significant environmental consequences [1,2]. Animal leather production relies heavily on animal slaughter, excessive water usage, and chemical treatments, generating toxic wastewater and soil contamination [3,4,5]. While synthetic leather eliminates animal use, it is derived from plastics and hazardous chemical additives, leading to non-biodegradable waste and long-term environmental issues [6]. Hence, there is an urgent demand for sustainable, eco-friendly alternatives.

Fungal mycelium leather, a novel bio-based material, offers renewable, biodegradable, and low-impact characteristics, addressing traditional leather’s challenges [7,8,9]. Mycelium is known for its rapid growth, adaptability, and efficient resource utilization, aligning with green manufacturing and sustainability goals [10,11]. The *Ganoderma lucidum* strain exhibits rapid growth and robust environmental adaptability. Its mycelium integrates three functionally specialized hyphae: generative (reproduction and differentiation), skeletal (structural reinforcement), and binding/ligative (nutrient transport), forming an interlocking network with enhanced mechanical properties, making it ideal for mycelium-based biomaterials [12]. Applications for mycelium leather span clothing, interior design, and packaging [13,14].

Existing mycelium production methods primarily rely on solid-state or liquid fermentation [15,16,17]. Solid-state fermentation employs agricultural residues as substrates to promote mycelial membrane growth [18]. While this method is simple and resource-efficient, it struggles with uniformity and scalability [19]. For instance, MycoWorks’ solid-state process faces challenges in achieving consistent film thickness and high output [20,21]. While liquid fermentation has been explored for mycelium growth through precise parameter optimization, and this mothed prevent hyphal aggregation and promote homogeneous biomass formation, addressing the key bottleneck of structural consistency in prior studies [22,23,24]. However, the dehydration and shaping of mycelial membranes remain key challenges [25]. The existing methods of mycelium dehydration, such as direct drying and freeze-drying, often compromise membrane quality by causing surface hardening, uneven porosity, or high energy consumption [26]. One approach to reduce water content involves the incorporation of fiber or organic waste into the fermentation broth, which aids in the formation of a mycelial membrane [27]. However, this practice deviates from the original aim of producing pure mycelium leather. Freeze-thaw cycling exploits ice crystal formation to facilitate intracellular water removal and reorganise mycelial structures into dense and ordered networks [28]. This approach not only maintains structural integrity, but also enhances mechanical properties and stability [29], thereby contributing to the development of mycelium leather with superior physical characteristics. Additionally, this method avoids the use of harsh chemicals or extensive processing steps, adhering more closely to the principles of sustainable and eco-friendly material production.

To sum up, this study aims to develop a novel, eco-friendly, and cost-effective method for the production of high-quality biomass materials. The liquid fermentation process provides a controlled environment for microbial growth, allowing for the precise manipulation of nutrient sources and growth conditions. Subsequently, the freeze-thaw dehydration step leverages the natural physical properties of ice crystal formation to enhance the structural characteristics of the resulting biomass. This study not only explores the feasibility of this integrated approach, but also evaluates its potential impact on the mechanical properties, stability, and overall performance of the biomass materials produced.

## 2. Materials and Methods

### 2.1. Materials

Glucose, agar, potassium dihydrogen phosphate, anhydrous magnesium sulfate, vitamin B_1_, glycerol, sodium hydroxide, and genipin were sourced from Sinopharm (Shanghai, China). Yeast powder and peptone were procured from Oxoid, Basingstoke, UK.

### 2.2. Experimental Procedures

The strains (*Ganoderma* spp. G1–G20) were obtained from the microbiological culture collection center of Jilin Agricultural University, Changchun, China. The activated strain was inoculated onto a plate containing PDA medium and incubated at 28 °C for 5 days.

#### 2.2.1. Seed Culture Preparation

The seed culture medium was prepared by dissolving glucose (3% *w*/*v*), peptone (0.5% *w*/*v*), yeast powder (0.25% *w*/*v*), potassium dihydrogen phosphate (0.01% *w*/*v*), magnesium sulfate (0.05% *w*/*v*), and vitamin B_1_ (0.005% *w*/*v*) in 1 L of deionized water. The mixture was stirred to obtain a homogeneous solution. The medium was then sterilized in an autoclave (HiClave HV-50, Shanghai, China) at 116 °C for 30 min. After cooling to 25 °C, five agar plugs (diameter: 0.5 cm) from pre-cultured *Ganoderma* spp. G15 plates were aseptically transferred into a 500 mL Erlenmeyer flask containing 200 mL of the sterilized medium. The flask was incubated in a rotary shaker (ZHWY-211B, Zhicheng, Shanghai, China) at 28 °C and 150 rpm under dark conditions for 7 days to obtain the seed culture.

#### 2.2.2. Liquid Fermentation

The fermentation medium was formulated by dissolving the following components in 3 L deionized water: glucose (3% *w*/*v*), peptone (0.5% *w*/*v*), yeast extract (0.25% *w*/*v*), KH_2_PO_4_ (0.01% *w*/*v*), MgSO_4_·7H_2_O (0.05% *w*/*v*), and thiamine hydrochloride (0.005% *w*/*v*). After complete dissolution through 1 h homogenization at 150 rpm, the medium underwent steam sterilization at 121 °C for 30 min. Following cooling to 25 °C, the seed culture was aseptically inoculated at a 10% (*v*/*v*) inoculation ratio. Fermentation was conducted in a 5 L stirred-tank bioreactor (Baoxing Biotech, Shanghai, China; Model BIOTECH-5BG) equipped with real-time dissolved oxygen and pH monitoring systems. The operational parameters were maintained as follows: temperature 28 ± 0.5 °C, agitation speed 150 rpm, aeration rate 1 vvm, and pH 5.5 ± 0.1, automatically regulated through 1 M NaOH/HCl addition. The 7-day fermentation process under dark conditions included periodic sampling for biomass quantification using gravimetric analysis.

#### 2.2.3. Freeze-Thaw Dehydration

The fermentation broth was subjected to centrifugation at 8000 rpm and 25 °C for 10 min to remove extracellular water. The centrifuged mycelium was transferred into a stainless steel mold and frozen at −7, −15, −40, or −80 °C for 48 h. After freezing, the mycelium was removed and allowed to thaw at room temperature in a stainless steel draining mold. Once thawed, the mycelium was returned to the same low-temperature environment for an additional 24 h of freezing. This freeze-thaw cycle was repeated three times. Finally, the unthawed mycelium membrane was removed, layered between five sheets of absorbent filter paper, and pressed under 1 MPa for pressure thawing. The membrane was then dried in an oven at 45 °C for 48 h to obtain the mycelium dry membrane. The samples subjected to freezing and thawing at −7, −15, −40, or −80 °C were designated as −7 FM, −15 FM, −40 FM, and −80 FM, respectively.

#### 2.2.4. Post-Treatment

**Scheme 1:**
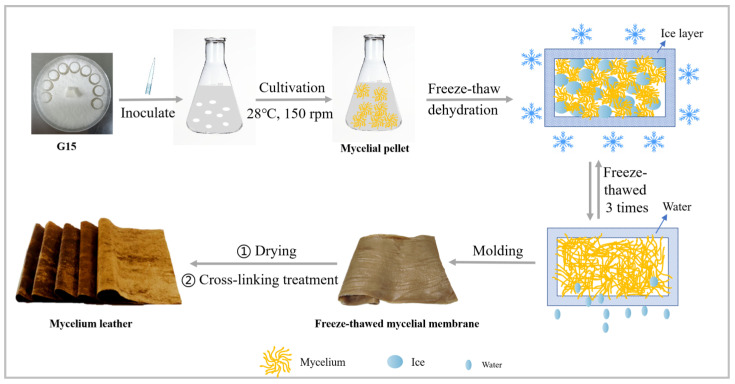
Diagram of mycelium leather preparation.

The harvested mycelium membrane (−15 FM) was soaked in a 20% sodium hydroxide (NaOH) solution for 6 h for alkali washing and deacetylation. The membrane was then rinsed three times with distilled water (30 min per rinse) and dried in an oven at 45 °C for 48 h. The dried membrane was immersed in crosslinking agent solutions containing genipin (0.5%, 1%, or 1.5%) or tannic acid (3%, 4%, or 5%) at 60 °C for 12 h [30]. Subsequently, the membrane was soaked in a 20% glycerol plasticizer solution for 24 h and dried at 30 °C for 24 h to obtain the final product, designated as liquid deep fermentation mycelium leather. The crosslinked membranes were labeled as follows: −15FLG-0.5%, −15FLG-1%, −15FLG-1.5%, −15FLT-3%, −15FLT-4%, and −15FLT-5%. The specific process is shown in Scheme 1.

### 2.3. Characterisation

#### 2.3.1. Mechanical Properties

The tensile strength of the mycelium leather was evaluated utilizing a WDW-5A electronic universal testing machine manufactured by Jinan Wenteng Testing Instrument Co., Ltd. (Jinan, China). The testing parameters were established as follows: a tensile speed of 200 mm·min^−1^, a controlled temperature of 25 °C, and a relative humidity of 50% [31]. The samples were prepared in dimensions of 100 mm × 10 mm, shaped in a dumbbell configuration. Each specimen underwent three repetitions of the test to ensure data reliability. The results are reported as averages, with standard deviations calculated to evaluate data consistency. The analysis adhered to the Chinese national standard GB/T 38610-2020 [32].

#### 2.3.2. Microstructure

The microstructural properties of the mycelium leather were examined by utilizing a scanning electron microscope (SEM) model (ZEISS SIGMA 300, Oberkochen, Germany). Prior to the analysis, the samples underwent a metal coating procedure to improve their conductivity. The experimental parameters included an acceleration voltage of 10 KV and a working distance of 10 mm. Additionally, the surface morphology of the samples was assessed using an atomic force microscope (AFM) model (Bruker Dimension Icon, Billerica, MA, USA) in tapping mode, with a scanning speed of 1 Hz and a scanning area of 2.5 × 2.5 µm, thereby ensuring high-quality and high-resolution imaging [33]. Through the application of these two microstructural characterization techniques, a comprehensive examination of both the surface and the internal structure of the mycelium leather was performed. Furthermore, a biological microscope (ZEISS Axio Lab.A1, Oberkochen, Germany) was employed to elucidate the morphological characteristics of ice crystals within the mycelial membrane, utilizing a cold light source illumination system to mitigate thermal damage to the specimens. Prior to this analysis, the samples were frozen in liquid nitrogen to maintain the integrity of the ice crystal morphology. Optimal image contrast and resolution were achieved by fine-tuning the focal length and aperture settings.

#### 2.3.3. Physical and Chemical Properties

The chemical bonds and functional group characteristics of the mycelium leather samples were analyzed using Fourier transform infrared spectroscopy (FTIR) with a Thermo Nicolet iS5 instrument (Waltham, MA, USA), which operated within a scanning range of 4000–500 cm^−1^ and conducted a total of 32 scans. In addition, the FTIR spectra were normalized by 1055 cm^−1^. Additionally, thermogravimetric analysis (TGA) was performed using a TGA-4000 apparatus (PerkinElmer, Waltham, MA, USA) to evaluate the thermal decomposition behavior and thermal stability of the samples. The static contact angle measurements were obtained by utilizing an XG-CAME device from Shanghai Xuan Zhi Chuang Xi Industrial Equipment Co., Ltd. (Shanghai, China).

#### 2.3.4. Self-Healing Ability

A self-healing experiment was conducted based on the existing literature [34]. The mycelium membrane, which has undergone freeze-thaw cycles and subsequent drying, is cut into a dumbbell shape. A hole with a diameter of 6 mm (Φ6 mm) is created in the central section using a hole punch. This prepared membrane is then placed in a 150 mm (Φ150 mm) Petri dish containing PDA culture medium, ensuring that appropriate humidity levels are maintained (Figure S1, shown in the Supporting Information; the same below). Subsequently, a sterile syringe is employed to uniformly inject 1 mL of malt extract broth (MEB) culture medium into the cross-section surrounding the central hole. The assembly is then incubated at a temperature of 28 °C, and observations are made to assess the viability of the mycelium membrane at various time intervals. It is imperative that all procedures, including mycelium culture, centrifugation, freeze-thawing, and drying, are performed under sterile conditions to prevent contamination.

#### 2.3.5. Moisture Content and Dehydration Rate

The moisture content and dehydration rate are calculated using Formulas (1) and (2), respectively, to assess the impact of the freeze-thaw process on the dehydration effect of the mycelium.

(1) $$M\;(\%)=\frac{m_{1}-m_{2}}{m_{1}}\times 100\%$$

*M*(%) is the moisture content, m_1_ is the initial mass of the wet mycelium (g), and m_2_ is the dry mass of the mycelium (g).

(2) V=100%−M(%)

In this equation, *V* represents the dehydration rate and *M* is the moisture content.

## 3. Results and Discussion

### 3.1. Liquid Fermentation and Dehydration

#### 3.1.1. Strain Screening

**Figure 1:**
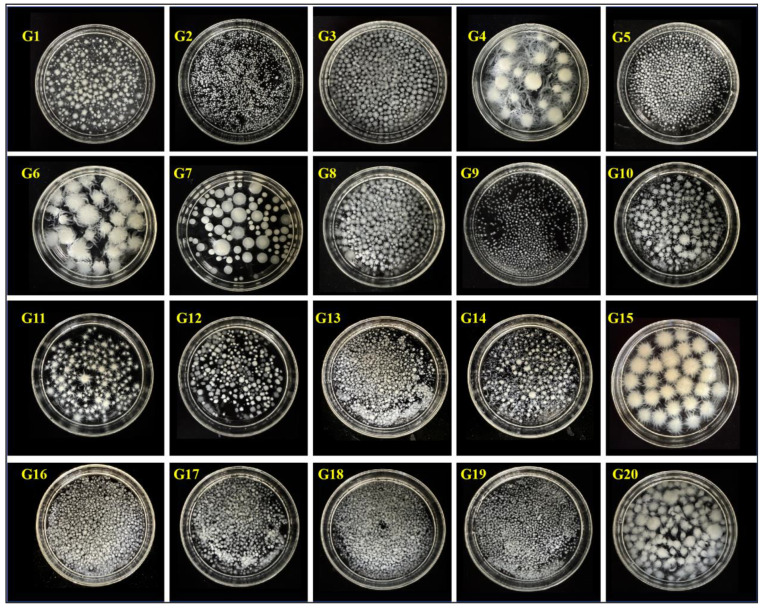
Morphological screening and biomass yield of 20 *Ganoderma* strains.

**Figure 2:**
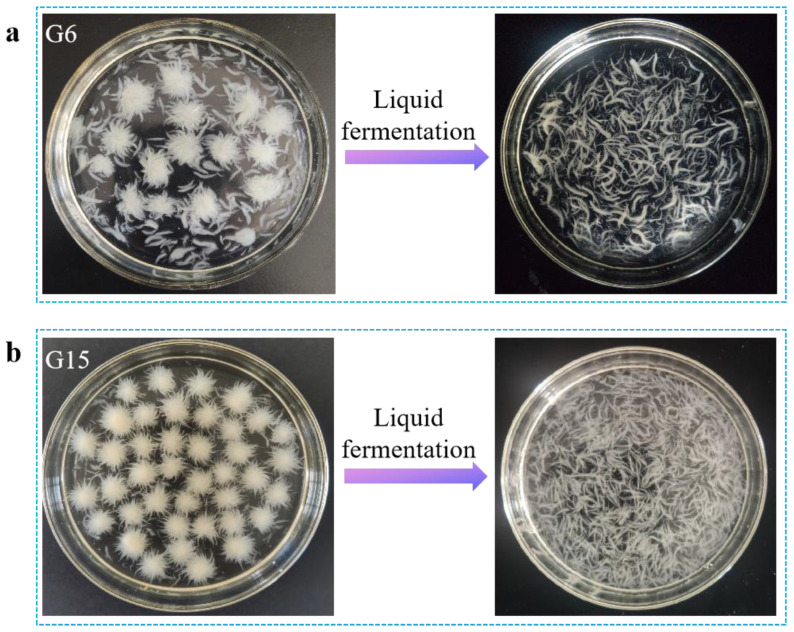
Photos of mycelial morphology of (**a**) G6 and (**b**) G15 before and after liquid fermentation.

To ensure a uniform texture in the mycelial leather material, 20 *Ganoderma* strains (Table S1) preserved in the laboratory were screened based on two key criteria: high biomass yield and consistent mycelial morphology. The selected strain was intended to serve as the core material for subsequent experiments, ensuring that the final mycelial leather met the desired standards in texture, strength, and appearance. As illustrated in Figure 1, the screening of 20 fungal strains under varying culture conditions revealed significant variations in colonial growth and morphology. Notably, strains G6 and G15 demonstrated uniform colonial morphology characterized by velvety spherical structures, accompanied by rapid mycelial development. This morphological consistency and accelerated growth pattern suggested their potential for higher biomass production, making them particularly suitable for mycelial fermentation. Consequently, these two strains were selected for a 7-day liquid fermentation culture. Post-cultivation observations (Figure 2) demonstrated a distinct morphological transition in both G6 and G15, with their cellular structures evolving from spherical forms to differentiated filamentous configurations. G15 developed fibrous mycelia measuring 5–8 mm with excellent uniformity, while G6 produced similar fibrous structures, but with lower consistency. Furthermore, G15 demonstrated the highest average biomass yield at 8.85 g·L^−1^ (Figure S2). Consequently, G15 was chosen as the optimal strain for further experimentation.

#### 3.1.2. Impact of Freeze-Thaw Parameters

**Figure 3:**
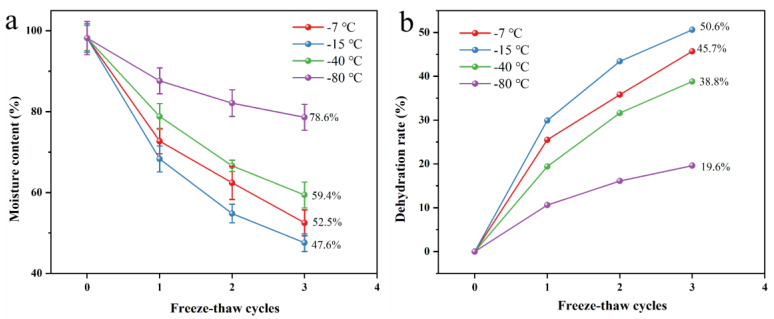
Water content (**a**) and dehydration rate (**b**) at −7 °C, −15 °C, −40 °C, −80 °C, respectively, under different freeze-thaw conditions.

The freeze-thaw process demonstrated significant enhancement of the dehydration efficiency of fungal mycelial membranes. As shown in Figure 3a, three freeze-thaw cycles induced a nonlinear reduction in water content with decreasing temperatures, reaching the minimum value (47.6%) at −15 °C. Interestingly, the water content unexpectedly increased to 59.4% and 78.6% at −40 °C and −80 °C, respectively, potentially due to ice crystal restructuring enhancing the water retention capacity. Figure 3b shows that the dehydration rate peaked at −15 °C (50.6%), with lower values observed at −7 °C (45.7%), −40 °C (38.8%), and −80 °C (19.6%). These findings establish −15 °C as the optimal temperature for achieving maximum dehydration efficiency, where the balance between ice crystal formation and membrane permeability likely facilitates effective water removal.

#### 3.1.3. Mechanism of Freeze-Thaw Dehydration

**Figure 4:**
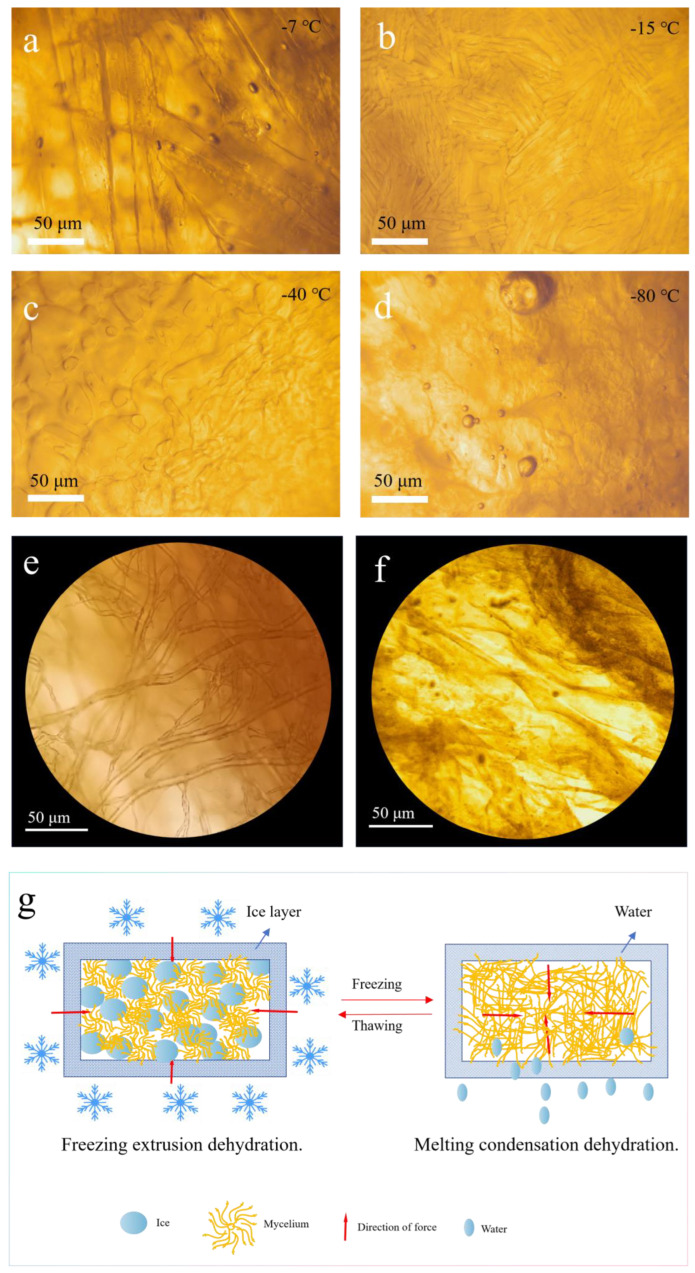
(**a**–**d**) Microscopic images of intracellular ice crystals in mycelial membrane under different freeze-thaw conditions; optical microscope images of mycelial morphology (**e**) before and (**f**) after freeze-thaw; (**g**) schematic of dehydration mechanism for mycelial membrane preparation under freeze-thaw conditions.

The formation and the behavior of ice crystals play a pivotal role in the freeze-thaw dehydration of fungal mycelium membranes [35]. During freezing, water molecules within the cells and extracellular spaces transition into ice, forming a lattice structure [36]. The size and the distribution of these ice crystals directly influence the dehydration efficiency and structural integrity of the material [37]. At −15 °C, the controlled cooling rate produced uniformly distributed and moderately sized ice crystals, as observed under a light microscope (Figure 4b). These crystals exert mechanical pressure on cell walls, causing micro-level ruptures that facilitate intracellular water migration. This process not only ensures effective water removal, but also induces the rearrangement of chitin molecules within the cell walls, enhancing the material’s structural stability. Conversely, at −7 °C, the ice crystals were elongated and unevenly distributed (Figure 4a), which resulted in less efficient dehydration and larger pore formation in the final structure. At lower temperatures, such as −40 °C and −80 °C, ice crystal formation was hindered due to rapid freezing (Figure 4c,d). In these conditions, intracellular water remained trapped, leading to incomplete dehydration and denser, less porous structures. Particularly at −80 °C, the lack of distinct ice crystal formation was noticeable, which significantly hindered water migration. Moreover, Figure 4e,f present optical microscope images of the mycelial morphology before and after the freeze-thaw cycles. As seen in the images, the mycelial fibers are intact and well-formed prior to the freeze-thaw process. However, after the freeze-thaw cycles, the fibers become unrecognizable, appearing fragmented. This suggests that the ice crystals formed during the freeze-thaw process disrupt the mycelial cell walls, leading to the breakdown of the fiber structure. This study proposes a potential mechanism for the freeze-thaw dehydration preparation of mycelial membranes (Figure 4g). During the freeze-thaw process, the formation and evolution of ice crystals plays a crucial role in the dehydration of mycelial cells. Initially, ice crystals form in the extracellular spaces, and as the temperature continues to decrease, the water inside the cells gradually transforms into ice. The growth of these ice crystals exerts mechanical pressure on the mycelial cell walls, causing deformation or damage. Additionally, the formation of intracellular ice crystals occupies space, applying mechanical stress to organelles and the cell membrane, which may lead to membrane rupture and deformation or damage to the organelles (Figure 4g, left side). As the ice crystals continue to grow, intracellular water migrates outward, increasing the concentration of intracellular solutes and creating a hypertonic environment. Water migration is driven by the chemical potential difference induced by the temperature gradient, with ice crystals preferentially forming in the extracellular space, raising the solute concentration outside the cell and further promoting the expulsion of intracellular water. In the melting phase, ice crystals transition into liquid water, but due to membrane damage caused by freezing, the cell’s ability to reabsorb water is impaired, leading to further expulsion of intracellular water (Figure 4g, right side). In the subsequent cold-pressing process, external pressure, combined with the pressure from ice crystal growth, facilitates the rearrangement of chitin molecules in the mycelial cell walls. Chitin, as the primary component of the fungal cell wall, undergoes rearrangement, altering the porosity and permeability of the cell wall, thus accelerating the expulsion of intracellular water.

### 3.2. Properties of Mycelial Membrane

#### 3.2.1. Microstructural Analysis

**Figure 5:**
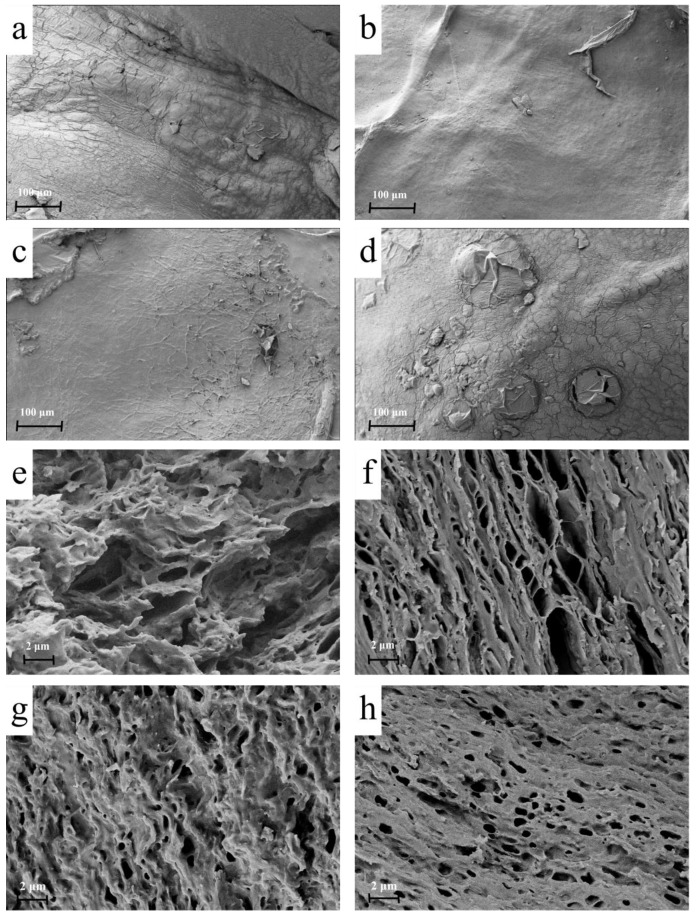
Surface and cross-sectional SEM images of (**a**,**e**) −7 FM, (**b**,**f**) −15 FM, (**c**,**g**) −40 FM, and (**d**,**h**) −80 FM.

The microstructure of the mycelial membrane after the freeze-thaw dehydration treatment is shown in Figure 5. At −7 °C (Figure 5a,e), large, irregularly distributed ice crystals formed, leading to an uneven pore structure. This resulted in weak mechanical properties due to the inconsistency of the membrane’s internal structure. At −15 °C (Figure 5b,f), the ice crystals were smaller and more uniformly distributed, producing a compact and layered network with uniform pores. This structural refinement optimised stress distribution within the membrane, contributing to improved tensile strength and elongation at break. In contrast, at −40 °C (Figure 5c,g), the rapid freezing caused amorphous ice crystal formation, disrupting the organised pore network and reducing porosity. At −80 °C (Figure 5d,h), the absence of visible ice crystals resulted in excessive compaction of the mycelium, leading to overly dense membranes with reduced flexibility and increased brittleness. The above results indicate that the freeze-thaw process significantly influences the microstructure of fungal mycelium membranes.

#### 3.2.2. Chemical Composition Analysis

**Figure 6:**
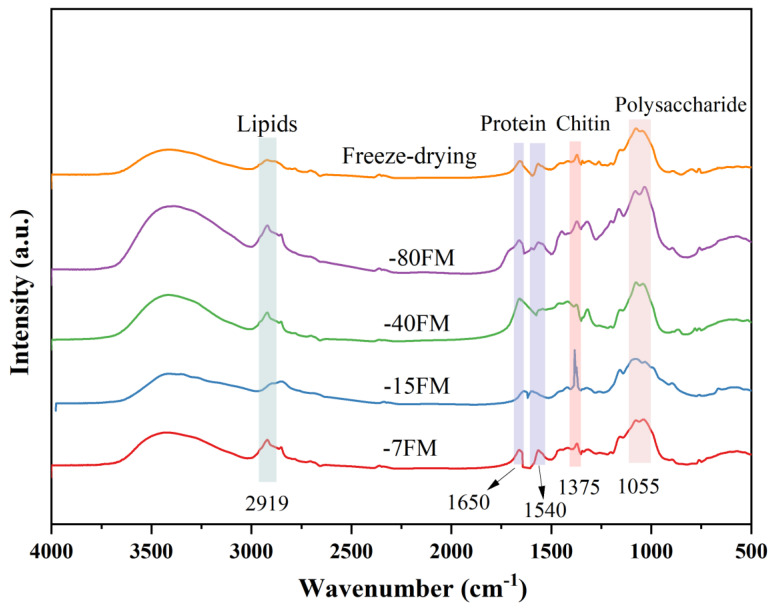
FTIR spectra of mycelial membranes.

During the freeze-thaw process, ice crystals rupture the cell walls, altering cell permeability. As water is expelled, intracellular substances are also released, resulting in changes in the composition of the mycelial membrane at different freeze-thaw temperatures. The chemical composition of the fungal mycelium membrane underwent notable changes across different freeze-thaw treatments, as confirmed by FTIR and TGA analyses. FTIR spectra (Figure 6) revealed distinct variations in the functional group distribution of the membranes, providing insight into the impact of freeze-thaw processing on the molecular structure. Key characteristic peaks included the following: 2919 cm^−1^: associated with the C-H stretching vibrations in lipids [38]; 1650 cm^−1^: corresponding to the amide I band, indicative of the α-helix structure in proteins [39]; 1540 cm^−1^: related to the amide II band, reflecting the β-sheet configuration of proteins [40]; 1375 cm^−1^: attributed to the stretching vibrations of chitin, a primary component of fungal cell walls [33,41]; 1055 cm^−1^: corresponding to the C-O stretching vibrations in polysaccharides [23]. A comparative analysis of these peaks demonstrated that the membranes treated at −15 °C exhibited significantly reduced intensities for lipid- and protein-related bands (2919, 1650, and 1540 cm^−1^), while the chitin-specific peak at 1375 cm^−1^ showed a marked increase. This suggests that the freeze-thaw process at −15 °C facilitated lipid and protein displacement and chitin reorganisation within the mycelium. Additionally, the intensity of the polysaccharide peak at 1055 cm^−1^ decreased, which may be due to the loss of some polysaccharides as water is expelled during the freeze-thaw cycling process.

TGA further validated the effects of freeze-thaw processing on thermal stability. As shown in Figure S3a, the decomposition process of the membranes can be divided into three temperature ranges. Low-temperature range (0–200 °C): mass loss in this range was primarily due to the evaporation of free and bound water [42]. Membranes treated at −15 °C exhibited lower mass loss compared to those treated at −7 or −40 °C, indicating more effective dehydration. Mid-temperature range (200–500 °C): this stage corresponded to the decomposition of organic compounds such as proteins, lipids, and chitin [43]. The −15 °C membranes showed slower decomposition rates, reflecting enhanced thermal stability, likely due to the increase in chitin content and reduced lipid content. High-temperature range (500–900 °C): minimal mass loss occurred in this range, which was attributed to the gradual breakdown of carbonaceous residues. The membranes treated at −15 °C displayed the most stable thermal profile, with smoother curves and lower residual mass compared to the other membranes. Differential thermogravimetric (DTG) analysis (Figure S3b) highlighted distinct peaks for organic component decomposition, with the −15 °C membranes exhibiting lower peak intensities and broader curves, indicating more robust thermal properties.

The integration of FTIR and TGA findings underscores the critical role of freeze-thaw conditions in modulating the chemical composition of fungal mycelium membranes. The enhanced chitin content, reduced lipid and protein levels, and improved thermal stability at −15 °C collectively contribute to superior mechanical properties and durability.

#### 3.2.3. Tensile Strength and True Density

The tensile strength [44] of the mycelial membranes under different freeze-thaw conditions is shown in Figure S4. At freeze-thaw temperatures of −7 °C, −15 °C, −40 °C, and −80 °C, the tensile strength levels of −7 FM, −15 FM, −40 FM, and −80 FM were 4.2, 4.5, 4.0, and 3.8 MPa, respectively. Correspondingly, their elongations at break were 11.70%, 15.37%, 12.70%, and 10.36%. These results indicate that −15 FM exhibited the highest tensile strength and elongation at break, which was attributed to the uniform distribution of pores and the reorganisation of chitin molecules, and this enhanced the material’s ability to distribute stress evenly.

True density measurements further corroborated these findings. As shown in Figure S5, the −15 °C membranes had the lowest true density (0.78 g·cm^−3^), consistent with their loosely packed and porous microstructure. The membranes treated at −7 °C had a moderate density compared to the other membranes (0.81 g·cm^−3^), indicating an optimal balance between porosity and structural integrity. Conversely, the membranes treated at −40 and −80 °C exhibited high true density, measuring 0.87 and 0.94 g·cm^−3^, respectively, reflecting their excessive compaction and reduced pore size, which adversely affected their flexibility and tensile properties.

#### 3.2.4. Comparison with Alternative Methods

**Figure 7:**
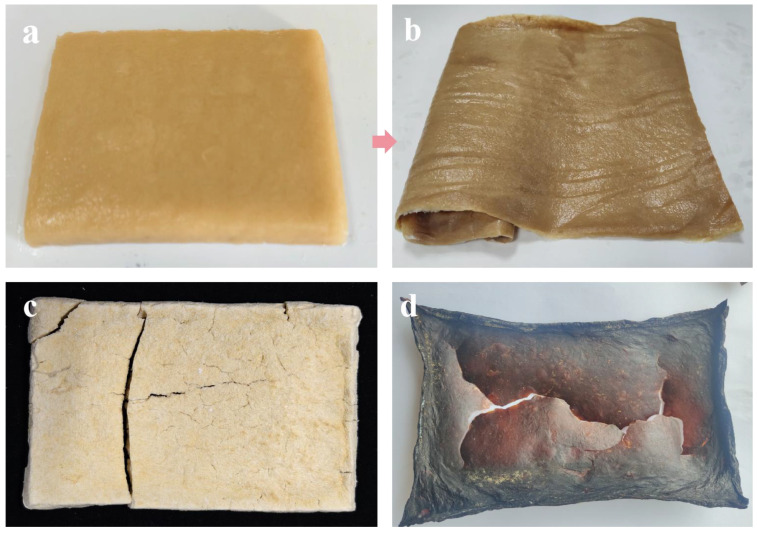
(**a**) Mycelial before freeze-thaw cycle dehydration, (**b**) mycelial membrane after freeze-thaw cycle dehydration, (**c**) mycelial membrane after freeze-drying dehydration, (**d**) mycelial membrane after high-temperature drying.

As shown in Figure 7a, after freeze-thaw dehydration the fungal mycelium presents a semi-solid gel-like texture with a smooth and uniform surface. This is due to the formation and melting of ice crystals during the freeze-thaw process, which allows the mycelium to be evenly distributed in the gel-like medium. This method not only offers high dehydration efficiency, but also effectively preserves the continuity and structural integrity of the material due to the mild processing conditions. The mycelial dry membrane (Figure 7b) is obtained by drying the mycelium after freeze-thaw dehydration. The dehydrated mycelial membrane exhibits high flexibility and continuity, providing favorable conditions for further mechanical processing. In contrast, the mycelium membrane obtained by freeze-drying (Figure 7c) appears block-like and loose in structure. This is because the freeze-drying process sublimates water under low temperature and pressure conditions, resulting in the formation of a porous structure that weakens the mechanical strength and processing adaptability of the membrane. Additionally, the mycelium membrane treated with high-temperature drying (Figure 7d) shows significant deformation and damage on the surface, and the color becomes darker. This is likely due to the rapid evaporation of water at higher temperatures during the drying process, which may have triggered certain chemical reactions (e.g., the Maillard reaction), thereby disrupting the structural continuity and uniformity of the membrane, making it unsuitable for further processing. Accordingly, the freeze-thaw dehydration method demonstrates clear advantages over conventional drying techniques such as freeze-drying and thermal drying.

### 3.3. Properties of Fungal Mycelial Leather

#### 3.3.1. Tensile Strength

**Figure 8:**
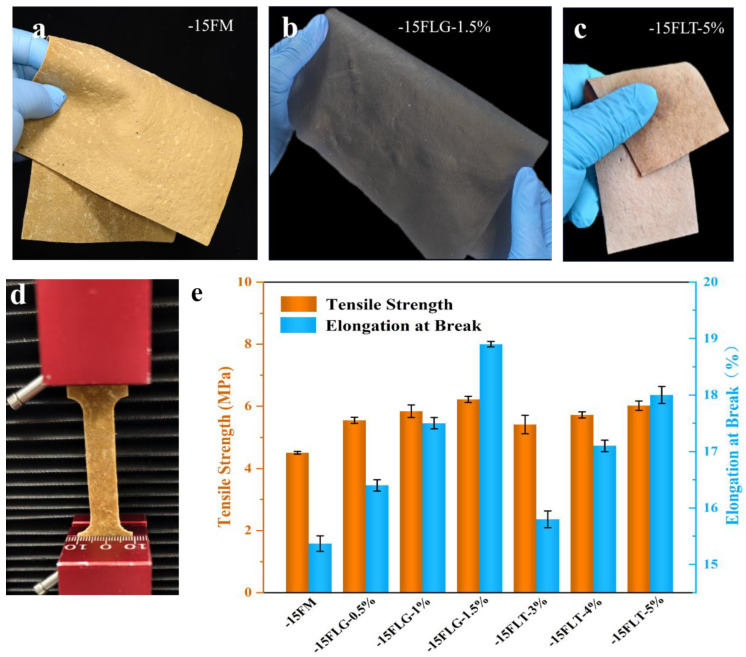
Images of (**a**) −15 FM, (**b**) −15FLG-1.5%, and (**c**) −15FLT-5%; (**d**) image of tensile strength testing; (**e**) tensile strength and elongation at break of different materials.

The −15 FM, which exhibited excellent properties, was crosslinked and pressed into fungal mycelium leather using genipin and tannic acid as crosslinking agents [45]. The pressed fungal mycelium leather exhibits good softness and elasticity (Figure 8a–c), a characteristic attributed to the tightly ordered network structure formed during the freeze-thaw dehydration process. Tensile tests (Figure 8d) show that the tensile strength of crosslinked fungal mycelium leather (−15FLG/−15FLT) is significantly increased (Figure 8e). Crosslinking also increased the elongation at break of the fungal mycelium leather, indicating improved flexibility and hand feel. Fungal mycelium leather crosslinked with genipin exhibits higher tensile strength than that crosslinked with tannic acid, due to the chemical reaction between the amino groups of chitosan and the aldehyde groups of genipin [46]. These aldehyde groups react with the amino groups to form stable imine bonds. Additionally, hydrogen bonding and molecular interactions between the hydroxyl and amino groups of chitosan and the functional groups in genipin further enhance the stability of the crosslinked structure [47]. The crosslinking mechanism between chitosan and tannic acid primarily involves hydrogen bonding and the reaction between phenolic hydroxyl groups and amino groups, which together facilitate the formation of the crosslinked structure. However, the crosslinking strength between chitosan and genipin is higher, primarily due to the multiple aldehyde groups and other reactive functional groups in the genipin molecule. These groups form stronger covalent bonds with chitosan, resulting in a higher crosslinking density and a more stable crosslinked structure. As the genipin concentration increased from 0.5% to 1.5%, the tensile strength and elongation at break of the fungal mycelium leather reached the maximum values of 6.22 MPa and 18.92%, respectively, which were higher than those of the pure white-rot fungi leather produced by J. Kniep et al. [24].

#### 3.3.2. AFM Analysis

**Figure 9:**
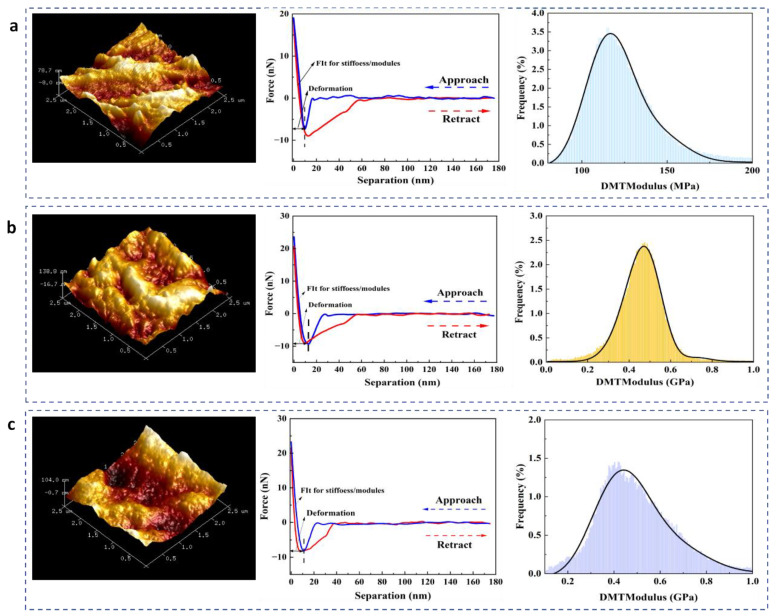
AFM images, force–separation curves, and DMT modulus distributions of (**a**) −15 FM, (**b**) −15FLG-1.5%, and (**c**) −15FLT-5%.

Atomic force microscopy (AFM) was used to characterise the microstructure and mechanical properties of −15 FM, crosslinked −15FLG-1.5%, and −15FLT-5% fungal mycelium leathers. The force–separation curves (Figure 9a–c) indicate that all the membranes display a three-dimensional structure and are elastic and deformable materials. Under forces of 20, 24, and 24 nN, the indentation depths of −15 FM, −15FLG-1.5%, and −15FLT-5% were 10.21, 12.82, and 11.54 nm, respectively, suggesting that crosslinked membranes have better resistance to deformation. Specifically, −15FLG-1.5% exhibited the greatest resistance. The hysteresis observed in −15 FM was significantly higher than that of the crosslinked membranes, indicating that −15 FM is softer and more deformable, with a weaker ability to recover its shape after deformation. The elastic moduli of −15 FM, −15FLG-1.5%, and −15FLT-5% were 116.16 MPa, 0.46 GPa, and 0.43 GPa, respectively. The crosslinked membranes exhibited much higher stiffness, confirming that crosslinking significantly improved the material’s mechanical properties. Fungal mycelium leather crosslinked with genipin showed superior tensile strength, elastic modulus, and shape stability, with reduced hysteresis. This demonstrates the potential of genipin as an efficient crosslinking agent for improving the mechanical properties of fungal mycelium leather.

#### 3.3.3. Water Contact Angle Analysis

**Figure 10:**
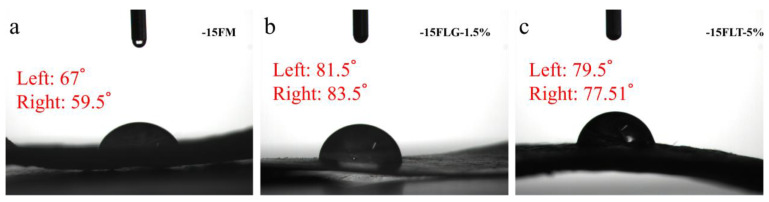
Water contact angles of (**a**) −15 FM, (**b**) −15FLG-1.5%, and (**c**) −15FLT-5%.

The water contact angle measurements revealed a notable increase in hydrophobicity after crosslinking (Figure 10a–c). Mycelium leather crosslinked with genipin (−15FLG-1.5%) exhibited a significantly higher water contact angle compared to tannic acid crosslinked samples (−15FLT-5%), indicating superior hydrophobicity. This enhanced surface property reflects the smoother and more uniform texture of the genipin-crosslinked leather. The improvement in surface smoothness can be attributed to the regulated arrangement of ice crystals during the freeze-thaw process, which promotes the formation of a dense and evenly layered mycelium structure. These findings underscore the role of genipin crosslinking in enhancing both the hydrophobicity and structural quality of fungal mycelium leather, making it a promising material for applications requiring water resistance.

#### 3.3.4. Self-Healing Capability Evaluation

**Figure 11:**
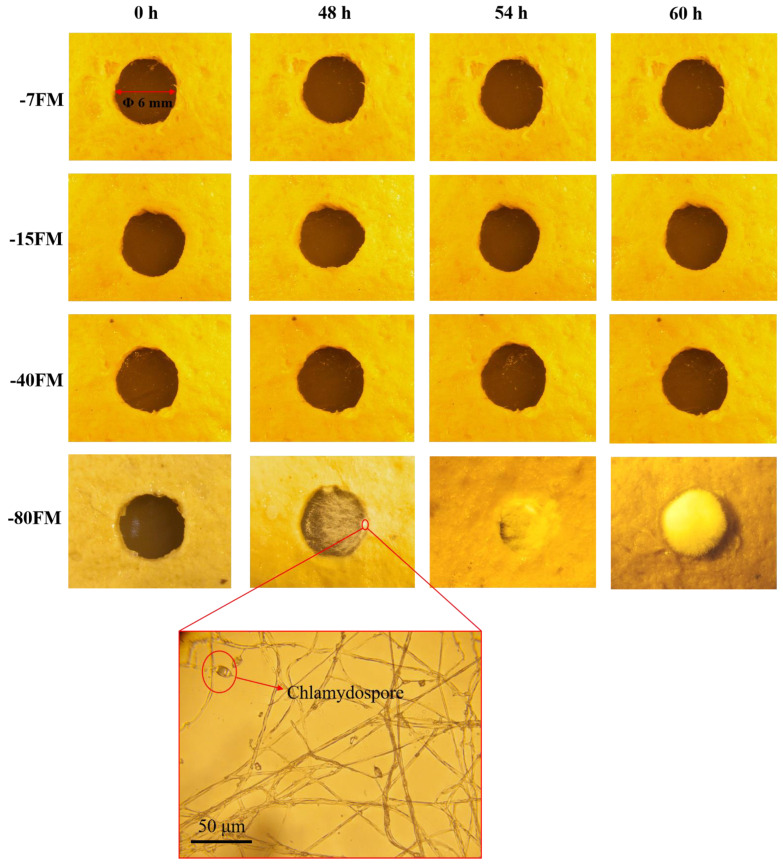
Optical microscope images of self-healing process of mycelial leather.

In the self-healing experiments, fungal mycelium leather samples were first cut into specific sizes to simulate potential damage in real-life applications [34]. The samples were then exposed to standard conditions to mimic typical damage scenarios. After cutting, the samples were placed in a controlled environment with a constant temperature and humidity, and their healing progress was observed at different time intervals. The results demonstrated that under controlled experimental conditions of −7, −15, and −40 °C, no new mycelial growth was detected in the areas exhibiting pore damage (Figure 11). Conversely, after 48 h of exposure to a temperature of −80 °C, new mycelial growth commenced and progressively covered the damaged areas over time (60 h). This observation suggests that the mycelial membrane subjected to freeze-thaw cycles at −80 °C retains a degree of physiological vitality, resulting in minimal cellular damage. However, following three freeze-thaw cycles, there was a significant decline in the vitality of the mycelium. As shown in the bottom image of Figure 11, optical microscopy revealed chlamydospores in the hyphae germinating at the pore edges at −80 °C. This phenomenon may result from the presence of chlamydospores within the interlayers of the mycelial membrane after three freeze-thaw cycles at −80 °C, which have the potential to germinate into new hyphae under favorable conditions (Figure S6). It is hypothesized that this retained physiological vitality is primarily attributable to the presence of chlamydospores situated within the mycelial membrane. Nonetheless, the repaired mycelial membrane exhibited an inadequate amount of mycelium in the intermediate pores, which was insufficient to completely fill the voids created by desiccation. When assessing tensile strength, the samples failed at the pore sites, yielding a measured strength of 1.22 MPa, which represents only one-third of the original tensile performance (Figure S7a,b).

#### 3.3.5. Ashby Plot Analysis

**Figure 12:**
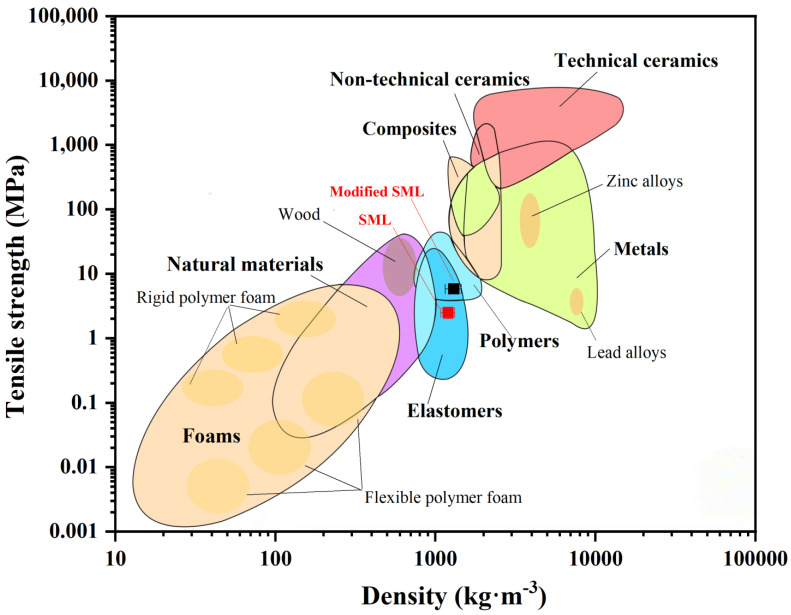
Density–strength Ashby plot of mycelium materials.

The Ashby plot, a powerful tool for material performance evaluation and comparison, is often used to construct coordinate systems based on key performance parameters [48]. For fungal mycelium leather, tensile strength and density were selected as the key parameters for the Ashby plot analysis. Figure 12 illustrates the relationship between tensile strength and density, including materials such as natural materials, polymers, composites, and technical ceramics. However, fungal materials are generally unsuitable for construction applications due to their insufficient structural support, as they typically consist of a single component. The −15 FM, −15FLG-1.5%, and −15FLT-5% membranes are categorized as mid-range materials, combining the characteristics of both elastomers and polymers due to their high density and tensile strength.

## 4. Conclusions

This study establishes a freeze-thaw dehydration method for advancing the production of mycelium leather through liquid fermentation. The optimized liquid fermentation protocol, incorporating precise pH control and dynamic agitation, enables homogeneous mycelial growth with enhanced structural consistency—a critical prerequisite for subsequent freeze-thaw processing. By synergizing controlled liquid-phase cultivation with ice-templated dehydration, this approach addresses the scalability and uniformity limitations inherent in traditional solid-state fermentation systems. The liquid fermentation environment facilitates the formation of uniform biomass matrices, which has proven essential for achieving ice crystal-mediated structural reorganization during dehydration cycles. Furthermore, the integration of fermentation parameter optimization with low-temperature processing underscores a novel pathway for balancing material performance with energy efficiency in biofabrication. This methodology not only demonstrates the pivotal role of fermentation design in mycelium material engineering, but also provides a scalable framework for industrial applications seeking to harmonize ecological sustainability with mechanical robustness. Future research directions include exploring fermentation–metabolite interactions to further enhance material functionality.

## Data Availability

The original contributions presented in this study are included in the article/Supplementary Materials. Further inquiries can be directed to the corresponding author.

## Supplementary Materials

The following supporting information can be downloaded at: https://www.mdpi.com/article/10.3390/jof11040326/s1, Figure S1: Self-healing cultivation experiment; Figure S2: Biomass of *Ganoderma* spp. G1–G20 strains after liquid fermentation; Figure S3: (a) TG and (b) DTG of −7 FM, −15 FM, −40 FM, and −80 FM; Figure S4: Tensile strength and elongation at break of −7 FM, −15 FM, −40 FM, and −80 FM; Figure S5: True density of −7 FM, −15 FM, −40 FM, and −80 FM; Figure S6: Microscopic image of the interlayers of the mycelial membrane after three freeze-thaw cycles at −80 °C; Figure S7: (a) Tensile fracture and (b) tensile strength of −80 FM after self-healing; Table S1: The used strain in the experiment.
