# Supplementary material for: Revolutionizing Eco-Friendly Leather Production: A Freeze-Thaw and Liquid Fermentation Approach with Fungal Mycelium
Source: J Fungi (Basel). 2025 Apr 19;11(4):326. doi: 10.3390/jof11040326 (PMC12028536; doi:10.3390/jof11040326)
Supplement: Supplementary file 1 [file jof-11-00326-s001.zip › jof-3537289-supplementary.pdf]

Support Information

Revolutionizing Eco-Friendly Leather Production: A Freeze-Thaw and Liquid Fermentation Approach with Fungal Mycelium

Linxin Song, Yuxin Liu, Shijun Xiao, Xiaohui Yuan, Xuerong Han*

International Cooperation Research Center of China for New Germplasm Breeding of Edible Mushrooms, Jilin Agricultural University, Changchun 130118, China

* Correspondence: hanxuerong@jlau.edu.cn

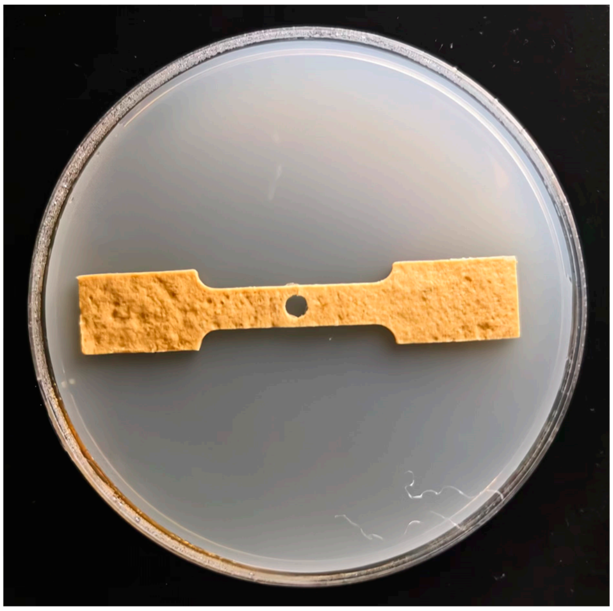

Figure S1. Self-healing cultivation experiment.

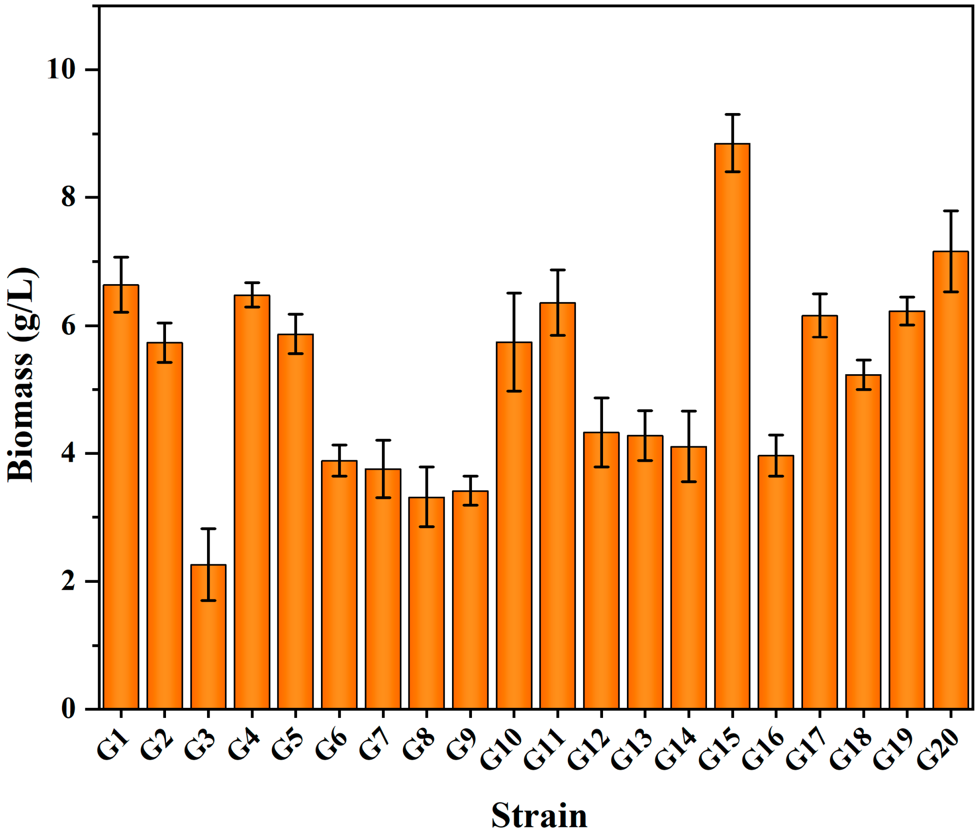

Figure S2. Biomass of *Ganoderma spp.*G1-G20 strains after liquid fermentation.

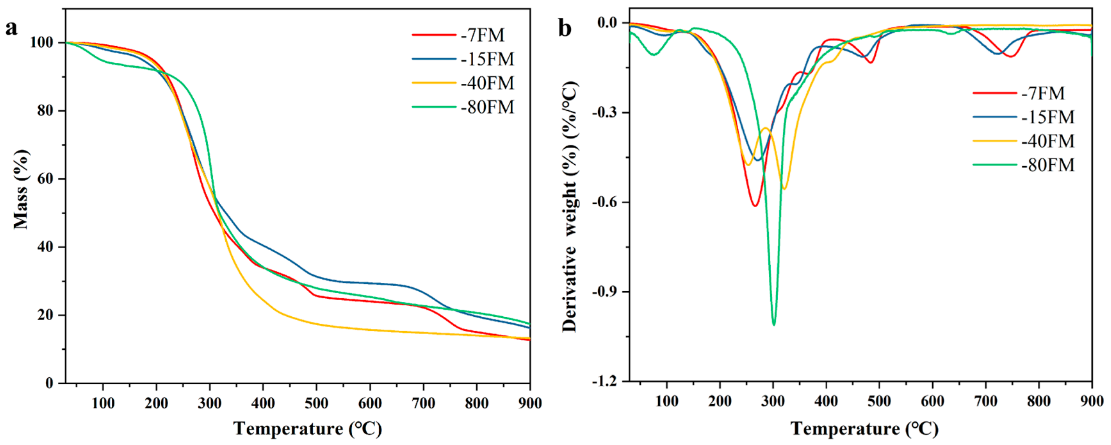

Figure S3. (a) TG and (b) DTG of —7FM, —15FM, —40FM, and —80FM.

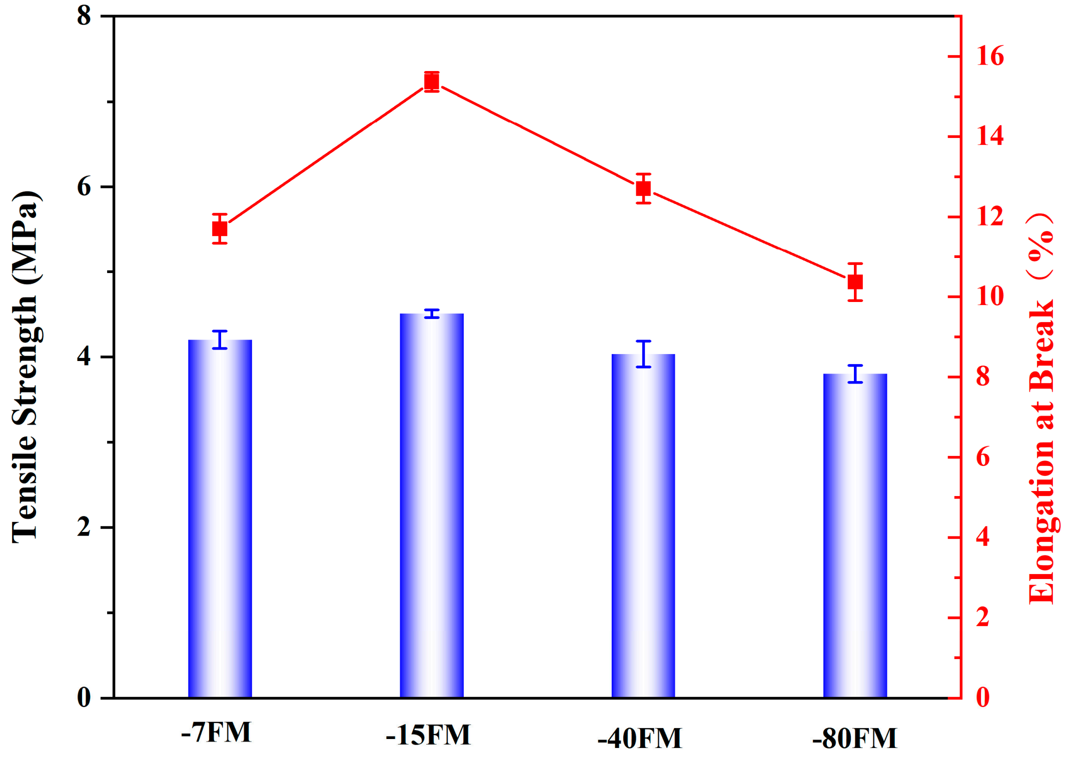

Figure S4. Tensile strength and elongation at break of -7FM, -15FM, -40FM, and -80FM.

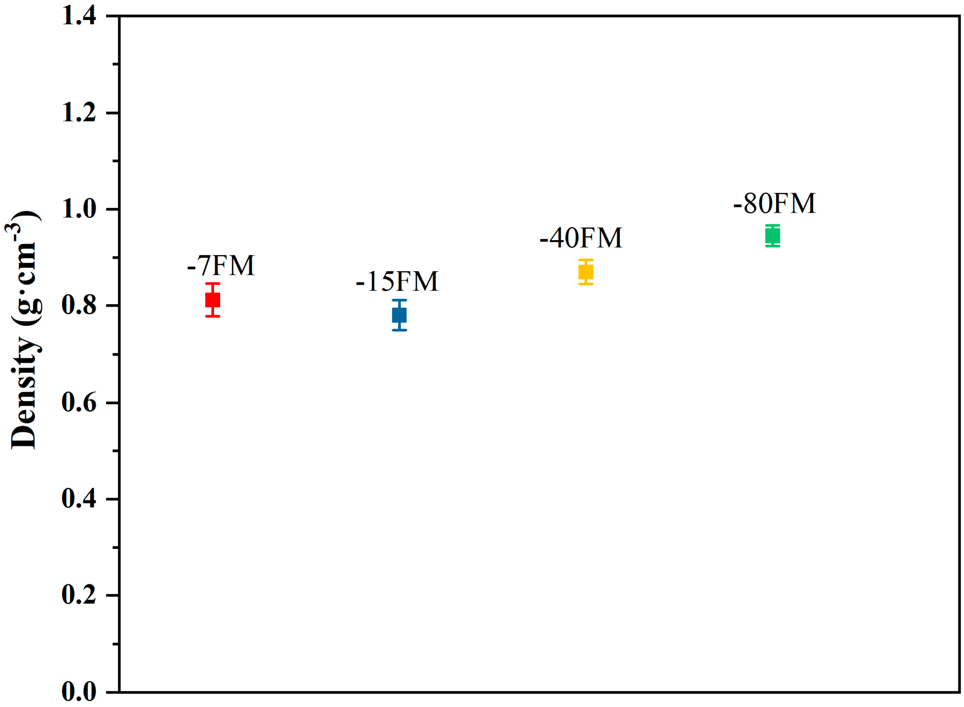

Figure S5. True density of -7FM, -15FM, -40FM, and -80FM.

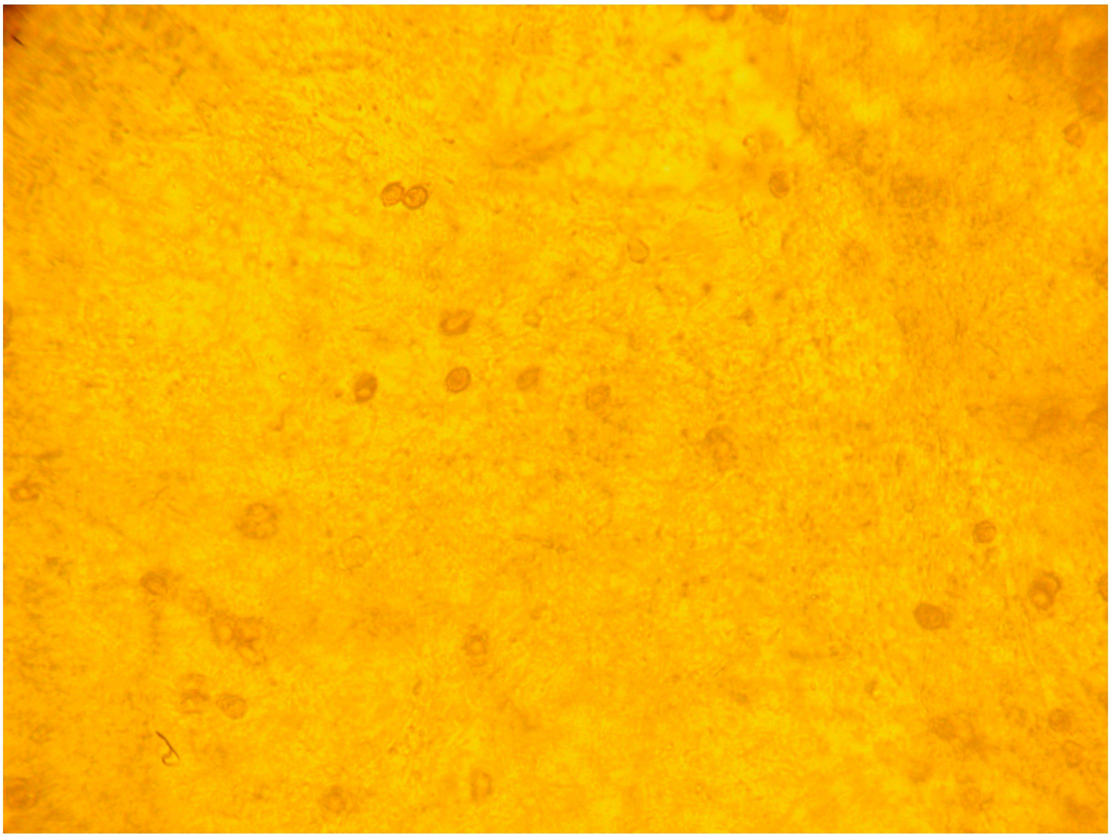

Figure S6. Microscopic image of the interlayers of the mycelial membrane after three freeze-thaw cycles at -80 °C.

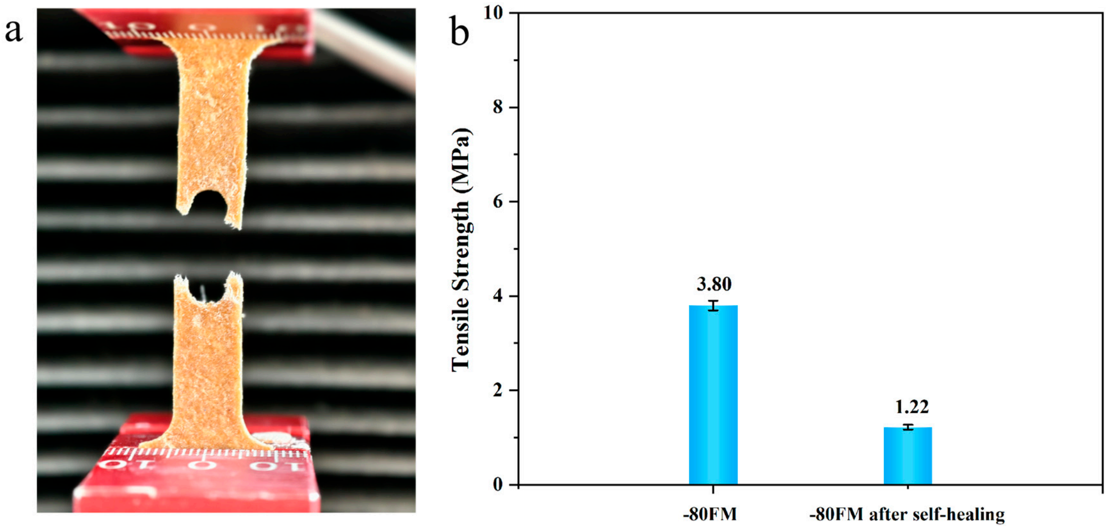

Figure S7. (a) Tensile fracture and (b) tensile strength of -80FM after self-healing.

Table S1 The used strain in the experiment.

Species	Strain number
G.lucidum	G1
G.lucidum	G2
G.lucidum	G3
G.lucidum	G4
G.lucidum	G5
G.resinaceum	G6
G.lucidum	G7
G.lucidum	G8
G.lucidum	G9
G.lucidum	G10
G.weberianum	G11
G.lucidum	G12
G.lucidum	G13
G.lucidum	G14
G.resinaceum	G15
G.lucidum	G16
G.lucidum	G17
G.lucidum	G18
G.lucidum	G19
G.lucidum	G20
